# Early detection of mild cognitive impairment utilizing ocular biomarker-based risk scoring nomogram

**DOI:** 10.3389/fnagi.2025.1669948

**Published:** 2025-12-03

**Authors:** Mohammed Seid Hussen, Bess Yin-Hung Lam, Wei Gao, Liping Zhou, Kai Yip Choi, Henry Ho-lung Chan

**Affiliations:** 1 Laboratory of Experimental Optometry (Neuroscience), School of Optometry, The Hong Kong Polytechnic University, Kowloon, Hong Kong SAR, China; 2 Department of Counselling and Psychology, Hong Kong Shue Yan University, Hong Kong Island, Hong Kong SAR, China; 3 Center for Eye and Vision Research (CEVR), New Territories, Hong Kong SAR, China; 4 School of Computer Science, University of Technology, Ningbo, China; 5 Research Centre for SHARP Vision (RCSV), The Hong Kong Polytechnic University, Kowloon, Hong Kong SAR, China; 6 Colour Imaging and Metaverse Research Centre, The Hong Kong Polytechnic University, Kowloon, Hong Kong SAR, China

**Keywords:** mild cognitive impairment, ocular biomarkers, risk scoring, dynamic nomogram, early detection

## Abstract

**Background:**

The prevalence of cognitive impairment is increasing along with global aging. Early retinal structural and vascular changes, prior to the onset of clinically detectable retinal pathologies, have been increasingly associated with cognitive changes. However, the evidence related to the predictive performance of these biomarkers remains limited. Therefore, this study aimed to develop and validate a nomogram-based scoring tool for opportunistic screening of mild cognitive impairment (MCI).

**Methods:**

This study prospectively recruited participants aged 60 years or older, including those with normal cognitive function. The retinal images were scanned using optical coherence tomography and angiography. Following the selection of potential predictors, a logistic regression model was built to predict MCI. Subsequently, a dynamic nomogram was developed to facilitate risk scoring in a clinical setting. The model’s discriminative ability was evaluated using the area under the receiver operating characteristic curve, along with diagnostic metrics of sensitivity and specificity at 95% confidence interval (CI). The model was internally validated using bootstrapping. Decision curve analysis was conducted to evaluate the model’s clinical impact and utility.

**Results:**

The model indicated that central macular thickness (*β*: −1.13; 95% CI: −0.15,-2.15; *p* < 0.05), outer nasal perfusion density in the macular area (β: 1.68; 95% CI: −2.92, −0.44; *p* = 0.008), and contrast sensitivity (β: −1.13; 95% CI: −2.03, −0.23; *p* < 0.05) were independently associated with MCI. This nomogram demonstrated a discriminative power of 0.90 (95% CI: 0.81, 0.98). The model also demonstrated good performance during bootstrap validation, achieving an AUC of 0.87. The optimal cutoff points achieved an accuracy of 86%, a sensitivity of 85% and a specificity of 87%. The decision curve analysis showed that the model provides a high net benefit.

**Conclusion:**

This study developed and internally validated a dynamic, nomogram-based scoring tool for early detection of MCI that integrates non-invasive retinal and visual biomarkers. The model demonstrated high discriminative power and substantial clinical net benefit. Further evaluation of the model’s prognostic value in predicting further cognitive decline may support its clinical utility.

## 1 Introduction

Mild cognitive impairment (MCI) is a transitional cognitive impairment with daily functioning largely preserved (Rodrigues and Moreno, 2023). Globally, the prevalence of MCI is estimated at 15–23% among older adults (Bai et al., 2022; Chen et al., 2023; Song et al., 2023; Salari et al., 2025). The underlying pathological changes may commence approximately a decade before the onset of mild cognitive changes, reflecting an asynchrony between structural and functional alterations that likely relate to neural compensatory mechanisms masking early deficits (Beason-Held et al., 2013; Reuter-Lorenz and Park, 2014). MCI is a pressing public health issue, characterized by a heterogeneous trajectory that may either progress to dementia with annual conversion rate of 7–16% (Tifratene et al., 2015; Roberts et al., 2014) or undergo reversion to normal cognitive function (Yu et al., 2025), which is orchestrated by a wide range of demographic, health-related, and biological factors. Higher risk of dementia is associated with higher age, cardiometabolic diseases, haemorrhagic stroke, depression, physical inactivity (Baik et al., 2025), higher body mass index (McGirr et al., 2022; Rosenberg et al., 2019), higher beta amyloid burden (Ottoy et al., 2019), and presence of tau (Smith et al., 2023; Bucci et al., 2021; Mendes et al., 2024). Vision impairment has also been recognized as a potential predictor that increases the risk of dementia in older adults, through limiting physical activity and social engagement (Shang et al., 2021; Lee et al., 2021). Age-related macular degeneration (AMD), glaucoma, and diabetic retinopathy have shown a significant association with an increased risk of all-cause dementia (Feng et al., 2023; Son et al., 2025; Shang et al., 2023). Predicting dementia based on these pathological changes may lack sensitivity for early prediction of cognitive decline, as it relies on pathological alterations rather than on preceding functional and structural disruption. Thus, objective biomarkers based on early structural and functional changes prior to the onset of pathological changes are the best strategy for early intervention. Given that the retina originates from the central nervous system and shares many structural and vascular traits (Lamb et al., 2007), several studies have investigated early retinal structural and vascular changes prior to the onset of clinically detectable retinal pathologies in AD and MCI. The studies reported reduction of retinal thickness (Sánchez et al., 2020; Cunha et al., 2017; Mei et al., 2021; Kim and Kang, 2019), retinal nerve fiber layer thickness (Szegedi et al., 2020; Yan et al., 2021), vessel density (Yoon et al., 2019; Wang et al., 2020; Ma et al., 2023), and perfusion density (Robbins et al., 2022; Ma et al., 2023). These retinal changes have been shown to correlate moderately with structural and functional brain changes (Hao et al., 2023; Wong et al., 2021). This indicated that retinal structural, vascular, and electrophysiological features are useful biomarkers that may signal the central neurovascular changes underlying early cognitive decline (Ge et al., 2021). Similarly, significant alterations in visual function have also been observed, including color vision deficiency (Cabrera DeBuc et al., 2018; Elvira-Hurtado et al., 2023; Salobrar-García et al., 2019; Vidal et al., 2022) and reduced contrast sensitivity (Risacher et al., 2013; Risacher et al., 2020). These early visual and retinal neurovascular changes might serve as cost-effective, non-invasive biomarkers for early detection of cognitive decline. However, the evidence related to the predictive performance of these biomarkers remains limited. Given that over one-third of ophthalmic patients are older adults aged 65 years or older (Guest et al., 1993), an integrated opportunistic screening program could be a potential strategy for this high-risk demographic. Thus, developing user-friendly risk assessment tools for this targeted population may facilitate early screening and bridge the gap between research findings and their practical application. Thus, transforming statistical models into nomograms provides an intuitive graphical scoring system that enhances the interpretability and applicability of the model by visually representing individual risk contributions (Jalali et al., 2019). Therefore, this study aimed to develop and validate a simplified clinical risk scoring tool for opportunistic screening of MCI.

## 2 Materials and methods

### 2.1 Study subjects

The Institutional Review Board of The Hong Kong Polytechnic University ethically approved this study. The study subjects underwent a comprehensive eye examination by licensed optometrists, in accordance with the principles outlined in the Declaration of Helsinki. Written informed consent was obtained from all subjects before the procedures. The inclusion criteria involved older adults aged 60 years or older, with best-corrected visual acuity equal to or better than 0.2 LogMAR, intraocular pressure of ≤21 mmHg, and refractive error of ≤±5.00 D. Subjects with a history of eye injury, dense cataract, glaucoma, AMD, diabetic retinopathy, or hypertensive retinopathy were excluded. Sample size adequacy was assessed using the clinical prediction model rule (Peduzzi et al., 1996). This study collected multiple retinal and visual features that could correlate with cognitive decline. However, most retinal features are highly correlated and lack unique predictive power for cognitive impairment. Hence, we considered retaining 4 to 5 predictors in the final model based on the following rationale: According to the clinical prediction model development rule (Stiell and Wells, 1999), a minimum of 5 and a maximum of 10 events are required for each predictor variable (Vittinghoff and McCulloch, 2007). Accordingly, a minimum of 15 and a maximum of 25 participants with MCI, as well as a comparable number of participants with NC, were required to fit the model. In this regard, our sample was adequate for developing the prediction model. Similarly, power analysis following the model fitting showed that nearly 51 samples were required to detect the desired effect size (section 3.5).

### 2.2 Cognitive assessments

The cognitive assessment process involved thorough history-taking and cognitive assessment (Petersen, 2016; McCarten, 2013). The participants were probed for evidence of cognitive concerns. A cognitive assessment was performed using the Cantonese version of the Mini-Mental State Examination (MMSE) (Chiu et al., 1994), administered by trained research personnel. MMSE assesses working memory, short-term memory, attention, concentration, language, visuospatial abilities, and orientation. The MMSE score ranges from 0 to 30. Following cognitive assessment, the study subjects were categorized as having MCI or being cognitively normal (NC) based on an MMSE score < 26 (Salis et al., 2023) and reported cognitive concerns. Participants with MCI and those with NC independently conduct daily activities.

### 2.3 Eye health examinations

Comprehensive eye examinations, including thorough history, visual function assessment, and retinal imaging, were conducted by an optometrist, who was an independent assessor blinded to the subjects’ cognitive status and research hypothesis.

#### 2.3.1 Visual function assessment

##### 2.3.1.1 Chromatic sensitivity

Chromatic sensitivity was evaluated using the tablet-based Rabin Cone Contrast Test (RCCT) (Innova Medical, Inc., United States) (White et al., 2023). The test consisted of a randomized series of letters selected from the British Standards Institution’s letter set, including letters with similar legibility (H, N, V, R, U, E, D, F, P, Z). The letters assigned for L- and M-cone subtend 1.22 LogMAR, whereas those used for S-cone contrast have a size of 1.34 LogMAR. The letters are displayed on a gray background with a luminance of 21.5 cd/m^2^, with decreasing steps of 0.16 log units toward the cone-contrast threshold (L- and M-cone: 27.5–1%; S-cone: 173–7%), robustly indicating the red, green, and blue sensitivities, respectively. During the test, a single letter appears at the center of the screen at a fixed visual angle and viewing distance of 75 cm (Cabrera DeBuc et al., 2018). The subject must select the letter seen from an adjacent 10-letter matching display.

##### 2.3.1.2 Contrast sensitivity

Contrast sensitivity was measured using the Mars contrast sensitivity test (Mars Perceptrix, Chappaqua, NY, United States) (Dougherty et al., 2005). This chart has eight rows of six letters, comprising 48 letters, designed to subtend a visual acuity of 1.38 LogMAR. The letters’ contrast ranges from 91% (−0.04 log units) to 1.2% (−1.92 log units), arranged in steps of 0.04 log units. During assessment, uniform illumination of 85 cd/m^2^ was adjusted, along with a proper testing distance of 50 cm, using near correction glass.

### 2.4 Retinal image acquisitions and extraction

#### 2.4.1 Optical coherence tomography

Macular retinal thickness (MRT) and peripapillary retinal nerve fiber layer (pRNFL) thickness were scanned using Heidelberg Spectralis HRA-OCT (Heidelberg Engineering, Heidelberg, Germany). MRT was obtained with a macula-centered acquisition based on the Early Treatment Diabetic Retinopathy Study (ETDRS) strategy, encompassing a total of 30^0^ (8.7 mm) with nine subfields: central 1 mm^2^, inner 3 mm^2^, (including inner superior, inner inferior, inner temporal, inner nasal, and outer) 6 mm^2^, (including outer superior, outer inferior, outer temporal and outer nasal). The pRNFL thickness was captured with an optic disk-centered scan covering 15° across seven subregions of interest: temporal superior, nasal superior, temporal inferior, nasal inferior, nasal, temporal, and global.

#### 2.4.2 Optical coherence tomography angiography

**Figure 1:**
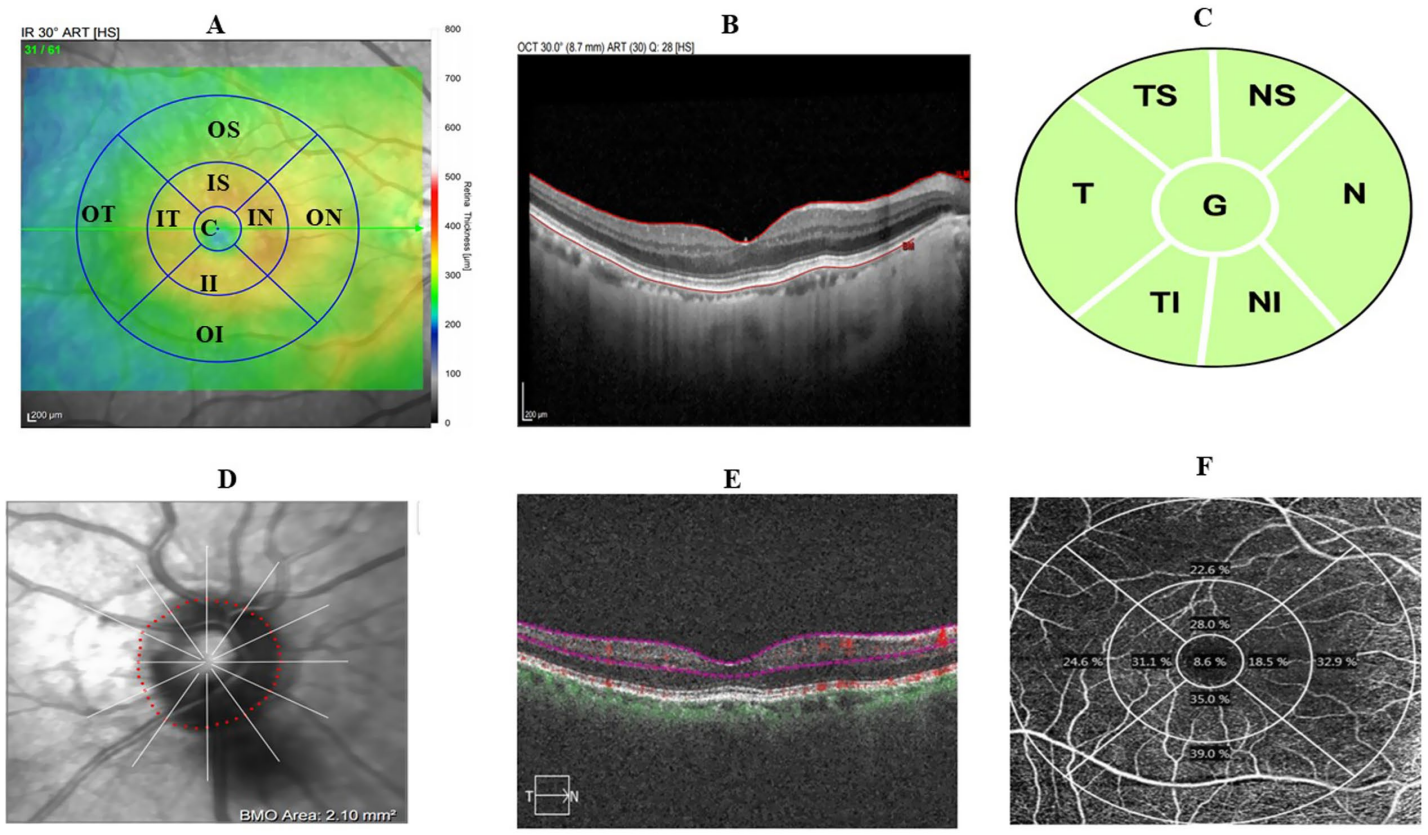
Retinal image scanning region based on the ETDRS grid. **(A)** Macular-centered scan showing 9 ETDRS grid. **(B)** Sample macular thickness segmentation. **(C)** Macular superficial vascular layer segmentation. **(D)** Sample macular superficial vascular layer map illustrating perfusion and vessel density. **(E)** Optic disc-centered scan of pRNFL thickness in 7 subregions of interest. **(F)** Sample optic disc-centered scan.

Optical coherence tomography angiography (OCTA) was performed using the spectral-domain Cirrus 6,000 (Carl Zeiss Meditec AG, Jena, Germany). The macular vessel density (MVD) and macular perfusion density (MPD) were scanned with a 6 × 6 mm^2^ scanning field. Images with a quality score of less than 7 and severely affected by motion artifacts were rescanned. The OCTA built-in algorithm segments the superficial vascular layer (SVL) from the inner boundary at the internal limiting membrane to the outer boundary of the inner plexiform layer. The MPD and MVD, along with the foveal avascular zone (FAZ) area, circularity (FC), and perimeter (FP), were extracted using the built-in algorithm (Figures 1A–F) (Samara et al., 2017; Laotaweerungsawat et al., 2020).

$$\text{Perfusion density}=\frac{\begin{array}{l} \text{Total area occupied}\;\mathrm{by}\;\\ \text{perfused vasculature}\;(\text{pixels}) \end{array}}{(\text{total scan area}-\mathrm{FAZ}\;\text{area})\;(\text{pixels})\;}$$

$$\text{Vessel density}=\frac{\text{Total length of skeletonized vasculature}\;(\mathrm{mm})}{\left(\text{Total scan area}-\mathrm{FAZ}\;\text{area})\;(\mathrm{mm}2\right)}$$

### 2.5 Data processing and analysis

#### 2.5.1 Predictor selection and model development

The collected data were checked for missing values and consistency. Retinal images taken from both were processed. Only two subjects had retinal imaging from only one eye due to poor image quality in the contralateral eye. The data collected were processed and analyzed. This, missing data was not observed at the subject-level. Following data processing, summary statistics were performed to characterize the visual, retinal, and cognitive profiles of the participants. Retinal and visual features were compared between participants with MCI and those with NC using an independent t-test and its nonparametric version, the Mann–Whitney test (*z*-test). A two-sided *p* < 0.05 was considered statistically significant. Predictors were screened using a generalized linear model (GLM) after adjusting for age. Multicollinearity was checked with a variable inflation factor (VIF). Potential predictors with less than 10 VIF were selected by the Least Absolute Shrinkage and Selection Operator (LASSO) regression model by using the “glmnet” R packages version 4.1–9^1^ and “MASS” R packages version 7.3–61,^2^ which penalizes less relevant features to zero coefficients and prevents overfitting (Ranstam and Cook, 2018).

Although there is no consensus on the ideal method for developing a model, simple models with fewer variables are usually preferred over complex models, as they are easier to interpret and apply in practice, despite excluding clinically important variables (Royston et al., 2009). Stepwise selection methods with a specific stopping rule or selection criteria are widely used, particularly in medical applications, to balance simplicity, retention of clinically relevant variables, and the risk of overfitting Since the stopping rule with a *p*-value of 0.05 is a stringent significance level that could potentially exclude clinically relevant factors, *p*-values with a cutoff of 0.1–0.2 are frequently recommended (Chowdhury and Turin, 2020). In this regard, the candidate predictors selected by LASSO were fitted into a binary logistic model using a backward stepwise method with a stopping rule of 0.1 to get the final model. Despite considering the assumption, we did not find any predictors retained in the final model with *p*-values between 0.05 and 0.1. Following fitting the final model, the Hosmer–Lemeshow goodness-of-fit test was performed at a *p*-value of 0.05. The model development process followed the guidelines for Transparent Reporting of Individual Prognosis or Diagnosis (Collins et al., 2015).

#### 2.5.2 Theoretical design

The theoretical design of the model was developed based on a literature review that has reported associations between retinal and visual changes and cognitive impairment.

Probability of MCI = f(predictor variables).

Pr (Y = 1) = f(x), where Y = 1, denotes presence of MCI (event), and Y = 0, indicate state of NC (no event).

Pr (Y = 1) = f(β0 + β1 age + β2 color vision+ β3 contrast sensitivity + β4 retinal thickness + β5 retinal vascular features).

*β*0 is the intercept term, representing the baseline log-odds of MCI when all predictor variables are zero. *β*1 through β5 are the coefficients corresponding to each predictor variable, quantifying the change in the log-odds of MCI associated with a one-unit change in the respective predictor.

#### 2.5.3 Nomogram construction

Based on the final model, an intuitive scoring tool, a nomogram, was developed. The scores of predictors in the nomogram were determined using the ratio method (Chen et al., 2022). The score of a predictor, denoted as *i*, is computed as:

$$\text{Score}\;i=(\mathrm{\beta i}*\text{observed value of predictor}\;i\;\text{after scalling})\frac{100}{\text{βmax}}$$

$$\begin{array}{l} \text{Observed value of}\;i\;\text{after scalling}=\text{maximum observed value of}\;i\\ -\text{Observed value of}\;i \end{array}$$

Where

βmax=βi∗maximumobserved value of predictoriafter scalling

where β is the regression coefficient of predictor *i*; $\frac{100}{\text{βmax}}$ is a scaling factor, βmax is the highest possible score of all predictors, and thereby the score of a predictor that provides βmax is set as 100. The total points were computed by summing the scores of each predictor. Furthermore, a dynamic nomogram app or web-based app was developed to simplify the application of the model in clinical settings. This web-based app was constructed using the “DynNom” version 5.1 and “rsconnect” version 1.5.1 functions in R to facilitate the individualized prediction in clinical practice. The “DynNom” package displays statistical model objects as a dynamic nomogram and generates the required scripts to publish it. The “rsconnect” function was used to deploy the built app to the Shiny web server at https://www.shinyapps.io/.

#### 2.5.4 Model performance evaluation and validation

The model’s diagnostic performance was evaluated using the area under the curve (AUC) computed from the receiver operating characteristic (ROC) curve, along with accuracy, sensitivity, specificity, positive predictive value (PPV), and negative predictive value (NPV). Model calibration was assessed by plotting deciles of predicted probability against the observed probability. The overall model performance was evaluated by the Brier score and Nagelkerke *R*-Square (*R*^2^), an adjusted version of the Cox and Snell R-Square (Nelson et al., 2016; Shabir et al., 2018). The Nagelkerke R-square ranges from 0 to 1, with higher values indicating better performance. The Brier score measures the accuracy of probabilistic predictions, ranging from 0 (perfect predictions) to 1 (worst possible predictions). The model was internally validated using bootstrapping to ensure its performance in future applications (Collins et al., 2015). Model validation by bootstrapping was performed by randomly generating bootstrap samples (*B* = 1,000) with replacement to evaluate the model’s optimism and optimism-corrected predictive performance. The optimism-corrected predictive performance of the model refers to the performance expected on unseen data. The “validate” and “val.pro” functions from the “rms” R package version 8.0–0^3^ were used to compare multiple model performance indices. The optimal risk score cutoff points were determined based on the Youden index method [(sensitivity + specificity) − 100%] that achieved the highest possible sensitivity and specificity across different cutoff points to evaluate the accuracy in the stratification of the study subjects into clinically distinct low- and high-risk categories.

#### 2.5.5 Decision curve analysis

Decision curve analysis (DCA) was used to evaluate the clinical impact of the model, a practical method that enhances clinicians’ decision-making (Vickers, 2008). Clinical decisions are usually navigated between two extreme situations: “treat all” and “treat none.” The specific intervention or treatment modality depends on the clinical context or problem under investigation. This study considered that retinal and visual features are risk indicators that facilitate further planning for patients attending an ophthalmic setting. The DCA compared the performance of retinal and visual features against two scenarios: “using cognitive tests for all” and “not using cognitive tests,” thereby capturing the consequences of decision-making based on a model that could yield false-negative or false-positive results. All statistical analyses, nomogram construction, and validation were performed using Stata/MP 17.0 (StataCorp LLC, 4095 Lakeway Drive, College Station, United States) and R 4.4.2 software^4^.

## 3 Results

### 3.1 Clinical characteristics of the study subjects

A total of 56 subjects participated: 30 with NC (mean age, 70.8 ± 7.7 years) and 26 with MCI (mean age, 77.9 ± 8.8 years). The mean cognitive score of subjects with MCI, as measured by the MMSE, was 20.34 ± 3.6, which was significantly lower than that of the NC subjects (28.08 ± 1.34, *p* < 0.0001). A significant proportion of the study subjects (75%) were females. Nearly half of the study subjects (51%) had chronic health conditions such as diabetes mellitus and hypertension. The L-, M-, and S-cone contrast thresholds were significantly reduced in subjects with MCI (*p* < 0.05). Significantly reduced contrast sensitivity was also observed in subjects with MCI (*p* = 0.0037). A significant sex-specific difference in cognitive score, contrast sensitivity, and chromatic contrast was not observed (*p* > 0.05) (Table 1).

**Table 1:** Clinical characteristics of the study subjects.

Variables	NC (*n* = 30)	MCI (*n* = 26)	*z*-test	*P*-value
Sex (F/M)	20/10	22/4	–	–
Age (years)	70.8 ± 7.7	77.9 ± 8.8	−2.838	**0.0045**
BCVA (logMAR)	0.05 ± 0.12	0.10 ± 0.13	−3.241	**0.0012**
IOP (mmHg)	13.57 ± 5.25	13.35 ± 4.15	1.003	0.3161
Mean MMSE	28.08 ± 1.34	20.34 ± 3.6	6.423	**<0.0001**
Diabetes mellitus (Yes/no)	4/26	4/22	–	**–**
Hypertension (Yes/no)	12/18	17/9	–	**–**
L-cone sensitivity	95 (87.5–102.5)	77.7 (67.7–87.7)	3.164	**0.0016**
M-cone sensitivity	90 (80–100)	75 (65–85)	3.634	**0.0003**
S-cone sensitivity	92.5 (87.5–97.5)	81.45 (68.95–93.9)	3.014	**0.0026**
Mean cone sensitivity	95 (88.34–101.67)	80.83 (70.83–90.83)	3.286	**0.0010**
Contrast sensitivity	1.64 (1.52–1.76)	1.44 (1.3–1.54)	2.861	**0.0037**

BCVA, best corrected visual acuity; IOP, intraocular pressure; MMSE, Mini-Mental State Examination. Bold values, statistically significant (*P* <0.05).

### 3.2 Comparison of retinal thickness-related features

Compared with NC subjects, MCI subjects showed a statistically significant reduction in MRT across all ETDRS retinal subfields, except the inner superior subfield. In contrast, for pRNFL, the temporal thickness was the only subfield that exhibited a significant decrease in subjects with MCI compared to the NC subjects (NC: 83.13 ± 12.69; MCI: 77.28 ± 12.77, *t* = 1.71, *p* = 0.046) (Table 2). Significant central retinal thickness within 3 mm diameter was noted in females compared to male participants (*p* < 0.05).

**Table 2:** Comparison of retinal thickness-related features between MCI and NC subjects.

Predictors	Retinal subfields	NC (*n* = 30)	MCI (*n* = 26)	*t*-test	*P*-value
MRT	Central	269.75 ± 43.5	260.25 ± 27.0	2.46	**0.0137**
	Inner superior	335.50 ± 22.5	330.00 ± 30.0	1.68	0.0921
	Inner inferior	335.75 ± 21.0	320.25 ± 25.5	2.33	**0.0196**
	Inner temporal	325.00 ± 29.5	315.75 ± 27.5	2.09	**0.0384**
	Inner nasal	342.50 ± 28.5	333.50 ± 27.5	2.07	**0.0384**
	Outer superior	295.95 ± 23.0	285.00 ± 18.0	2.42	**0.0153**
	Outer inferior	285.75 ± 31.5	270.25 ± 16.5	3.04	**0.0024**
	Outer temporal	297.25 ± 38.5	267.75 ± 19.0	2.44	**0.0143**
	Outer nasal	315.50 ± 21.50	300.25 ± 21.0	2.97	**0.0029**
	Volume	8.525 ± 0.77	8.21 ± 0.51	2.67	**0.0072**
MPD	Central	24.25 ± 11.0	18.63 ± 11.4	0.18	0.85
	Inner superior	23.42 ± 8.24	18.82 ± 8.65	2.03	**0.023**
	Inner inferior	23.77 ± 7.69	17.52 ± 8.80	2.83	**0.0032**
	Inner temporal	21.87 ± 7.77	17.15 ± 8.94	2.11	**0.0195**
	Inner nasal	22.3 ± 9.35	17.05 ± 7.35	2.26	**0.0138**
	Outer superior	27.98 ± 7.59	21.07 ± 8.02	3.30	**0.0008**
	Outer inferior	28.74 ± 8.63	20.16 ± 8.35	3.76	**0.0002**
	Outer temporal	24.25 ± 11.0	18.62 ± 11.4	2.6	**0.0044**
	Outer nasal	33.48 ± 8.94	22.85 ± 9.18	4.37	**<0.0001**
MVD	Central	2.75 ± 3.15	4.05 ± 3.25	−0.69	0.49
	Inner superior	10.92 ± 3.40	10.48 ± 3.31	0.43	0.33
	Inner inferior	11.30 ± 3.27	10.06 ± 4.44	1.12	0.11
	Inner temporal	10.875 ± 5.95	10.15 ± 3.8	0.52	0.60
	Inner nasal	17.03 ± 10.03	17.45 ± 9.32	−0.16	0.56
	Outer superior	13.38 ± 5.25	13 ± 4.85	0.88	0.37
	Outer inferior	13.43 ± 5.65	12.35 ± 4.3	1.54	0.12
	Outer temporal	11.23 ± 3.12	9.77 ± 3.88	1.55	0.06
	Outer nasal	16.5 ± 4.7	14.2 ± 5	2.04	**0.04**
pRNFL thickness	Nasal inferior	104.40 ± 17.49	112.5 ± 23.0	−1.49	0.90
	Nasal	76.13 ± 11.28	77.69 ± 13.8	−0.46	0.67
	Nasal superior	110.97 ± 25.48	117.7 ± 26.1	−0.98	0.83
	Temporal superior	140.28 ± 26.34	130.0 ± 29.5	1.96	0.05
	Temporal	83.13 ± 12.60	77.28 ± 12.8	1.71	0.046
	Temporal inferior	153.8 ± 25.90	157.1 ± 19.6	−0.53	0.70
	Global	100.35 ± 12.13	100.4 ± 13.5	−0.04	0.50

pRNFL, peripapillary retinal nerve fiber layer; MRT, macular retinal thickness; MVD, macular vessel density; MPD, macular perfusion density. Bold values, statistically significant (*P* <0.05).

### 3.3 Comparison of retinal vascular features

Compared with NC subjects, subjects with MCI demonstrated statistically significant reductions in MPD across all ETDRS subfields, except the central subfield. Notably, outer nasal MPD decreased more in MCI subjects than in NC subjects (*p* < 0.0001). Similarly, outer superior and inferior MPD were significantly lower in subjects with MCI compared to the NC subjects (CI: 21.07 ± 8.02; NC: 27.98 ± 7.59, t = 3.30, *p* = 0.0008) and (MCI: 20.16 ± 8.35; NC: 28.74 ± 8.63, t = 3.76, *p* = 0.0002), respectively. However, a significant reduction in MVD was observed only in the outer nasal subfields in subjects with MCI, and this was statistically lower than in NC subjects (*p* = 0.04). The outer temporal subfield also showed a trend toward reduction in subjects with MCI compared to the NC subjects, although this difference was not significant (*p* = 0.06) (Table 3). Significant sex-specific difference in retinal vascular changes was not observed (*p* > 0.05).

**Table 3:** Comparison of retinal vascular features of the superficial vascular layer between subjects with MCI and NC.

Predictors	Retinal subfields	NC (*n* = 30)	MCI (*n* = 26)	*t*-test	*P-*value
MPD	Central	24.25 ± 11.0	18.63 ± 11.4	0.18	0.85
	Inner superior	23.42 ± 8.24	18.82 ± 8.65	2.03	**0.023**
	Inner inferior	23.77 ± 7.69	17.52 ± 8.80	2.83	**0.0032**
	Inner temporal	21.87 ± 7.77	17.15 ± 8.94	2.11	**0.0195**
	Inner nasal	22.3 ± 9.35	17.05 ± 7.35	2.26	**0.0138**
	Outer superior	27.98 ± 7.59	21.07 ± 8.02	3.30	**0.0008**
	Outer inferior	28.74 ± 8.63	20.16 ± 8.35	3.76	**0.0002**
	Outer temporal	24.25 ± 11.0	18.62 ± 11.4	2.6	**0.0044**
	Outer nasal	33.48 ± 8.94	22.85 ± 9.18	4.37	**<0.0001**
MVD	Central	2.75 ± 3.15	4.05 ± 3.25	−0.69	0.49
	Inner superior	10.92 ± 3.40	10.48 ± 3.31	0.43	0.33
	Inner inferior	11.30 ± 3.27	10.06 ± 4.44	1.12	0.11
	Inner temporal	10.87 ± 5.95	10.15 ± 3.80	0.52	0.60
	Inner nasal	17.03 ± 10.03	17.45 ± 9.32	−0.16	0.56
	Outer superior	13.38 ± 5.25	13.00 ± 4.85	0.88	0.37
	Outer inferior	13.43 ± 5.65	12.35 ± 4.30	1.54	0.12
	Outer temporal	11.23 ± 3.12	9.77 ± 3.88	1.55	0.06
	Outer nasal	16.50 ± 4.70	14.2 ± 5.00	2.04	**0.04**

pRNFL, peripapillary retinal nerve fiber layer; MRT, macular retinal thickness; MVD, macular vessel density; MPD, macular perfusion density. Bold values, statistically significant (*P* <0.05).

### 3.4 Predictor selection using LASSO regression analysis

**Figure 2:**
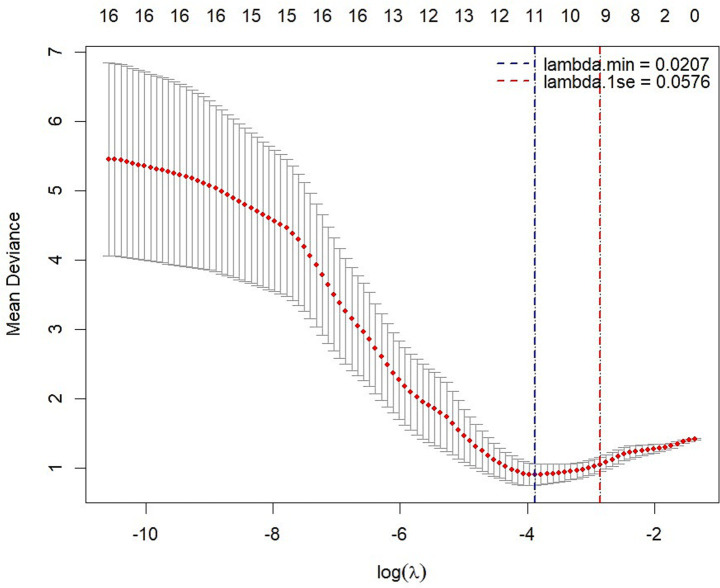
LASSO regression analysis with 10-fold cross-validation with a minimum error criterion to determine the optimal penalization estimate of *λ*.

Following screening for multicollinearity, 16 potential variables were examined using univariate analysis after adjusting for age (Table 4). These predictors were entered into the LASSO regression algorithm using a 10-fold cross-validation approach. Systematically, less relevant variables were shrunken to zero as the penalty coefficient (*λ*) increased. At the 0.056 optimal lambda value selected by cross-validation, 9 predictors, including age, contrast sensitivity, M-cone contrast, central MRT, outer temporal MRT, nasal inferior pRNFL, nasal superior pRNFL, outer nasal MPD, and outer superior MPD, were selected. In contrast, the coefficients of 7 predictors were shrunken to zero (Figure 2).

**Table 4:** Age-adjusted univariate analysis using a generalized linear model to identify potential predictors (*n* = 56).

Predictors		β coefficients (95% confidence interval)	*P*-value
MRT	Central	−0.91 (−1.74, −0.877)	**0.032**
	Inner superior	−0.62 (−1.30, 0.058)	0.073
	Inner inferior	−0.86 (−1.75, 0.019)	0.055
	Inner temporal	−0.72 (−1.44, −0.02)	0.045
	Inner nasal	−0.61 (−1.25, 0.03)	0.063
	Outer superior	−0.44 (−1.06, 0.18)	0.166
	Outer inferior	−0.95 (−1.86, −0.05)	**0.039**
	Outer temporal	−0.76 (−1.49, −0.04)	**0.039**
	Outer nasal	0.21 (−1.27, 0.23)	0.177
	Volume	−1.2 (−2.36, −0.05)	0.041
pRNFL thickness	Nasal inferior	0.63 (−0.088, 1.36)	0.085
	Nasal superior	0.47 (−0.14, 1.08)	0.130
	Temporal	−0.51 (−1.16, 0.13)	0.122
MPD	Inner inferior	−0.60 (−1.32, 0.105)	0.095
	Outer superior	−0.79 (−1.59, 0.002)	0.051
	Outer inferior	−0.99 (−1.84, −0.15)	**0.021**
	Outer nasal	−1.33 (−2.34, −0.33)	**0.009**
MVD	Inner nasal	0.73 (−0.021, 1.48)	0.057
Visual Function	L-cone sensitivity	−0.043 (−0.095,0.008)	0.10
	M-cone sensitivity	−0.06 (−0.12, −0.002)	0.04
	S-cone sensitivity	−0.06 (−0.13, 0.006)	0.073
	Contrast sensitivity	−3.84 (−7.32, −0.36)	**0.031**

pRNFL, peripapillary retinal nerve fiber layer; MRT, macular retinal thickness; MVD, macular vessel density; MPD, macular perfusion density. Bold values, statistically significant (*P* <0.05).

### 3.5 Model development using multivariate logistic regression analysis

The selected variables were further analyzed using multivariate logistic regression analysis. The selected predictors were fitted into a multivariable binary logistic regression model using a backward stepwise method with a stopping rule of 0.10 to identify independent predictors of MCI, yielding the final model. Consequently, the final model retained 3 predictors: central MRT, outer nasal MPD, and contrast sensitivity. The Hosmer-Lemeshow goodness-of-fit test yielded a *p*-value greater than 0.05, indicating that the model fits the observed data well. The model indicated that a one-unit decrease in central MRT (*β*: −1.13; 95% CI: −0.15,-2.15; *p* < 0.05) and contrast sensitivity (*β*: 1.13; 95% CI: −2.03, −0.23; *p* < 0.05) was associated with a 1.13-fold increase in the risk of MCI. Similarly, a one-unit reduction in outer nasal MPD (*β*: 1.68; 95% CI: −2.92, −0.44; *p* = 0.008) was associated with an increase in the risk of MCI by 1.68 (Table 5). Based on the final regression model, the risk score ranged from −9.52 to 7.73. Subjects with MCI had a mean risk score of 1.52 ± 2.02, which was significantly higher than the mean risk score of −1.84 ± 2.38 in subjects with NC (*p* < 0.0001). The probability of MCI was 0.43 when central MRT (276 μm), outer nasal MPD (28.55), and contrast sensitivity (1.52) were all at their mean. When the central MRT increased by one standard deviation above the mean (318 μm), while outer nasal MPD (28.55) and contrast sensitivity (1.52) remained at their mean, the probability of MCI was 0.2. Similarly, increasing contrast sensitivity (1.72) by one standard deviation above the mean, while keeping the central MRT (276 μm) and the outer nasal MPD (28.55) at their means, yielded a probability of MCI of 0.20. Likewise, the probability of MCI was 0.13 when outer nasal MPD (38.99) was increased by one standard deviation above the mean, with central MRT (276 μm) and contrast sensitivity (1.52) at their mean. The corresponding effect size for increasing central MRT and contrast sensitivity by one standard deviation above the mean, while keeping other predictors at their means, is 0.23. The predictors showed 0.2 non-significant correlation to each other. Hence, the retrospective power analysis indicated that nearly 51 samples were required to detect the desired effect size of 0.23.

**Table 5:** Multivariable binary logistic regression analysis to predict MCI (*n* = 56).

Predictors	Multivariable binary logistic regression	
	β coefficients (95% confidence interval)	*P*-value
Outer nasal MPD	−1.68 (−2.92, −0.44)	**0.008**
Central MRT	−1.13 (−2.15–0.15)	**0.029**
Contrast sensitivity	−1.13 (−2.03, −0.23)	**0.014**
Intercept	−0.28 (−1.01, 0.45)	0.453

MRT, macular retinal thickness; MPD, macular perfusion density. Bold values, statistically significant (*P* <0.05).

### 3.6 Nomogram construction

**Figure 3:**
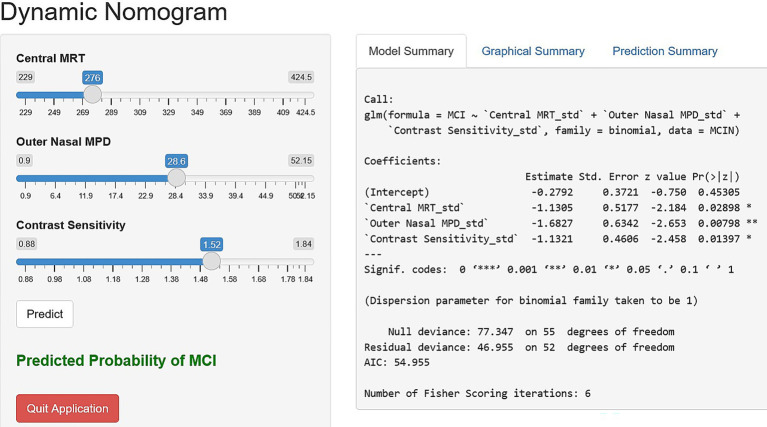
Dynamic nomogram application interface using central MRT, outer nasal MPD, and contrast sensitivity to simplify the risk scoring in clinical settings.

Based on the final regression model, a nomogram incorporating central MRT, outer nasal MPD, and contrast sensitivity was constructed using the “rms” package to predict the occurrence of MCI among older adults. Construction of the nomogram involved computing the total score and the prediction index scores for each variable using the ratio method. The risk contribution of each predictor at a given observed value was scalled between 0 to 100 points, and the sum of prediction index scores for each variable contributed to the total score. Higher total scores indicated a higher risk of MCI. Furthermore, a dynamic nomogram app was also developed using the “DynNom” packages to visualize statistical models. Subsequently, the “rsconnect” function was run to deploy on a web server at https://mciprediction.shinyapps.io/MCIAPP1/. This web-based calculator has a user-friendly interface that allows manipulation of the predictors’ observed values and computation of the corresponding predicted probability of MCI (Figure 3).

### 3.7 Evaluation and validation of the nomogram

**Figure 4:**
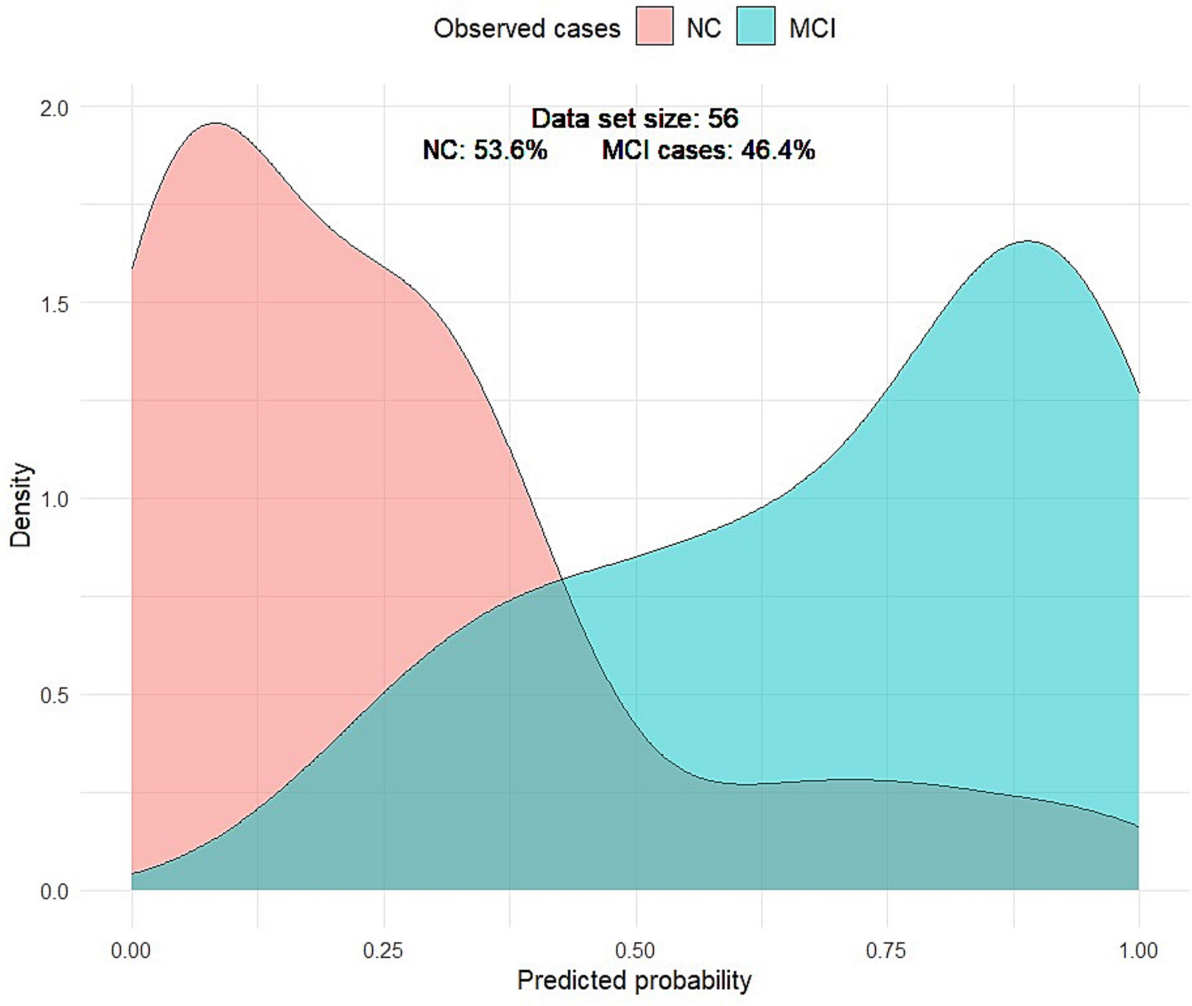
Density plot of predicted probabilities by MCI. The light red curve represents the density distribution for the NC group, and the light green curve represents the density distribution for the MCI group. The gray-green curve represents the false-positive and false-negative cases.

Visually, the density plot against predicted probabilities was bimodal, with two distinct peaks, reflecting the concentration of observed cases across two distinct probability ranges. Low probabilities indicate NC, and higher predictive probabilities represent MCI. This bimodality indicated that the model performed well in distinguishing MCI from NC (Figure 4).

**Figure 5:**
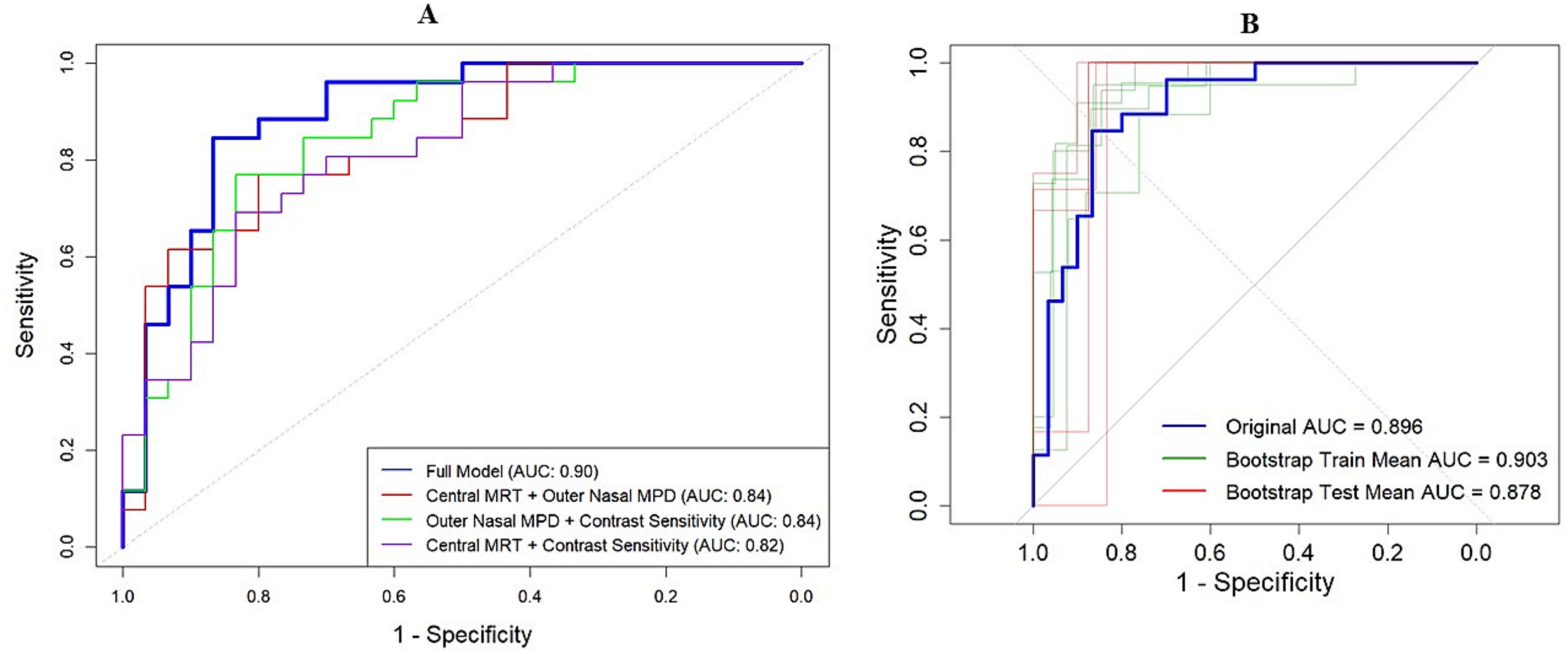
Model discrimination power indicated by AUC. **(A)** Model discrimination in the original dataset. The red curve represents AUC by combining outer nasal MPD and central MRT. The green curve indicates the AUC by combining nasal MPD and contrast sensitivity, while the pink curve denotes the AUC by combining central MRT and contrast sensitivity. The bold blue curve represents the AUC of the full model for detecting MCI among older adults. **(B)** Model discrimination in the bootstrap training (green curve) and testing (red curve) dataset compared to the original AUC (blue curve).

The nomogram-based model, which integrated central MRT, outer nasal MPD, and contrast sensitivity, demonstrated high discriminative power with an AUC of 0.896 (95% confidence interval: 0.81, 0.98). Alternatively, models combining only outer nasal MPD and contrast sensitivity, as well as outer nasal MPD with central MRT, also achieved a discriminative power of 0.84 (95% confidence interval: 0.74, 0.94) and 0.837 (95% confidence interval: 0.73, 0.94), respectively (Figure 1A). Through bootstrapping validation (B = 1,000), the bias-corrected AUC was 0.87, with an optimism value of 0.029, indicating that high discrimination power was also maintained for unseen data (Figures 5A,B).

**Figure 6:**
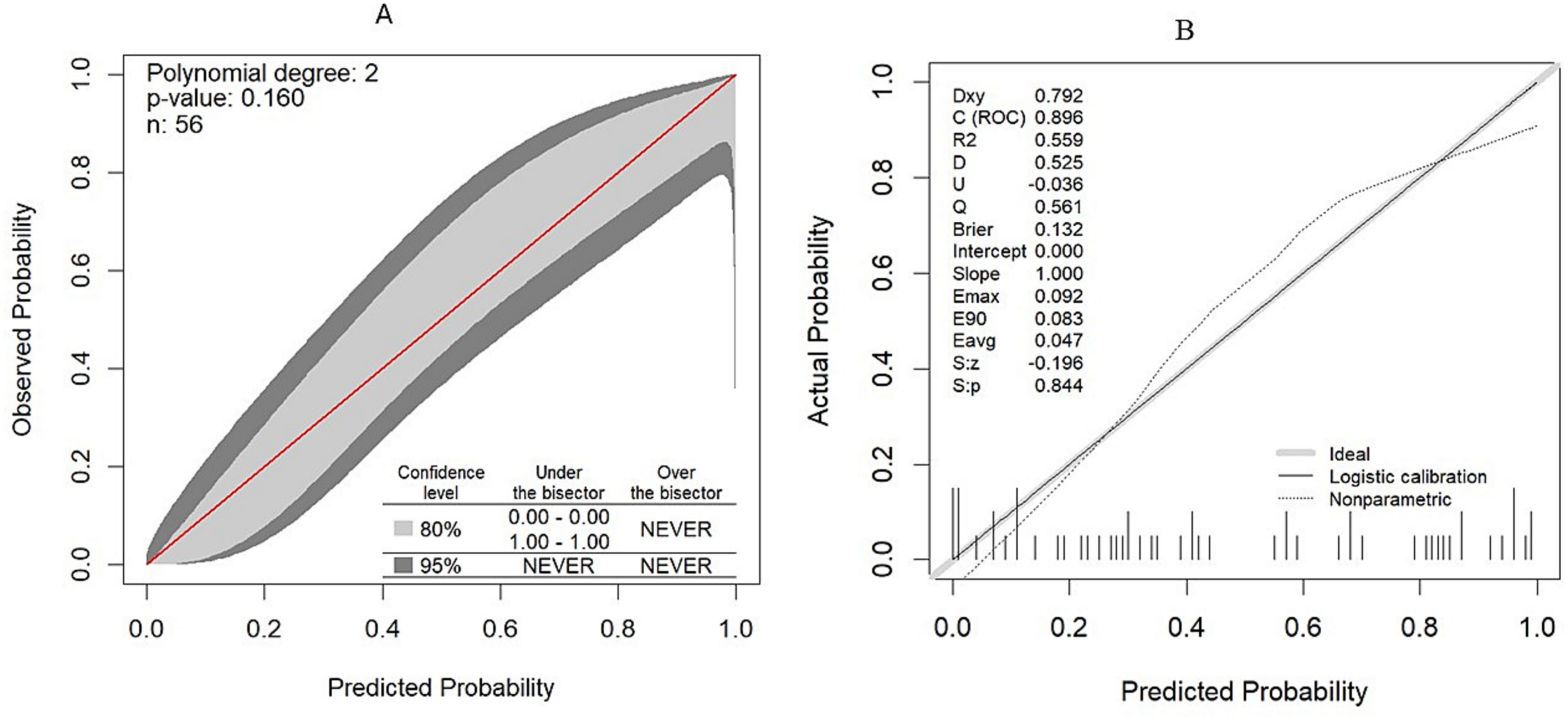
Model calibration plot. **(A)** Model calibration plot in the original dataset. **(B)** Model calibration plot after validation with the bootstrapping technique.

The calibration plot also showed no significant deviation, as the actual calibration curve (the gray-shaded area) was evenly bisected by the ideal calibration curve (the red line), indicating no significant under- or over-confidence, with a 95% confidence interval (*p* > 0.05). Following bootstrap validation, the model also showed no evidence that the bias-corrected calibration line deviates significantly from the ideal 45-degree line (*Z* = −0.196, *p* = 0.844), indicating good calibration with the test dataset (Figures 6A,B).

The overall model evaluation demonstrated reasonably high performance, with a Nagelkerke *R*-squared of 0.40. Spiegelhalter’s *z*-statistic *p*-value of the final model was 0.58, indicating that the observed and predicted probabilities were well matched and calibrated. The Brier score was 0.13, indicating high predictive accuracy. The optimism coefficients for the intercept, Brier score, and R2 were −0.0228, 0.06, and −0.0015, respectively, indicating the closeness of the bias-corrected value to the original index and ensuring high performance after validation (Table 6).

**Table 6:** Model performance after validation with the bootstrapping method in detecting MCI among older adults (*B* = 1,000).

Metric	Original index	Training	Test	Optimism	Corrected index
Dxy	0.7923	0.7972	0.7621	0.0351	0.7573
C (AUC)	0.8962	0.902	0.873	0.029	0.8672
R^2^	0.5594	0.5935	0.5312	0.0623	0.4971
Intercept	0	0	−0.0015	0.0015	−0.0015
Slope	1	1	0.8528	0.1472	0.8528
Emax	0	0	0.0358	0.0358	0.0920
D	0.5248	0.5795	0.4901	0.0894	0.4354
U	−0.0357	−0.0357	0.0565	−0.0922	0.0565
Q	0.5606	0.6152	0.4335	0.1817	0.3789
B	0.1319	0.1222	0.1450	−0.0228	0.1547
g	3.0158	3.8173	2.8184	0.9989	2.0169
gp	0.3898	0.3925	0.3782	0.0143	0.3755

Dxy, Somer’s D index; C(AUC), area under curve; *R*^2^, Nagelkerke *R* Square; B, Brier score; Emax, maximum difference between raw predicted probabilities and the recalibrated probabilities; D, Discrimination index; U, Unreliability index; Q, Overall quality; g = g-index (Gini’s mean difference); gp, 𝑔 index on the probability scale.

### 3.8 Optimal cutoff point determination

**Figure 7:**
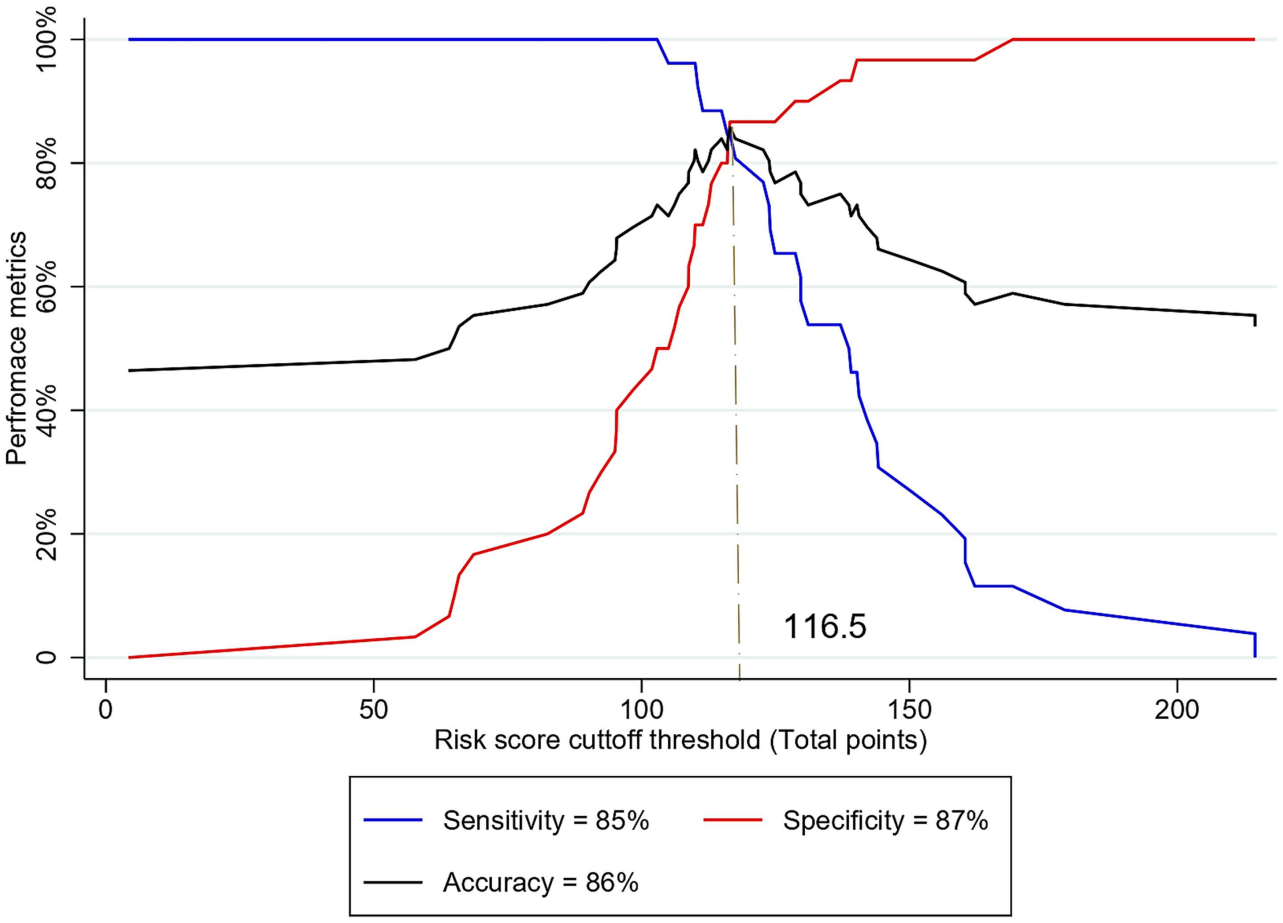
Sensitivity analysis of different cutoff thresholds of risk score based on the total points to predict MCI among older adults.

The optimal cutoff point for stratifying subjects into low- and high-risk categories for MCI, based on the model, was determined through sensitivity analysis using the Youden index method (Table 7). At a Youden index of 0.72, the optimal cutoff points of risk score and total points derived from the nomogram were −0.31 and 116.5, respectively. These optimal cutoff points achieved the highest possible accuracy of 85.71%, sensitivity of 84.62%, specificity of 86.67%, PPV of 84.6%, and NPV of 86.6% (Figure 7).

**Table 7:** Risk score value and corresponding estimated predicted probability of MCI, along with common performance indices of risk score-based classification.

Cutoff points (≥)	Sensitivity	Specificity	Accuracy	LR+	LR−
4.216	100.00%	0.00%	46.43%	1	
57.72	100.00%	3.33%	48.21%	1.0345	0
64.06	100.00%	6.67%	50.00%	1.0714	0
65.04	100.00%	10.00%	51.79%	1.1111	0
68.63	100.00%	16.67%	55.36%	1.2	0
82.43	100.00%	20.00%	57.14%	1.25	0
89.01	100.00%	23.33%	58.93%	1.3043	0
90.22	100.00%	26.67%	60.71%	1.3636	0
92.46	100.00%	30.00%	62.50%	1.4286	0
95.34	100.00%	40.00%	67.86%	1.6667	0
98.44	100.00%	43.33%	69.64%	1.7647	0
101.90	100.00%	46.67%	71.43%	1.875	0
105.00	96.15%	50.00%	71.43%	1.9231	0.0769
108.72	96.15%	60.00%	76.79%	2.4038	0.0641
110.00	96.15%	70.00%	82.14%	3.2051	0.0549
110.50	92.31%	70.00%	80.36%	3.0769	0.1099
112.48	88.46%	73.33%	80.36%	3.3173	0.1573
113.00	88.46%	76.67%	82.14%	3.7912	0.1505
114.90	88.46%	80.00%	83.93%	4.4231	0.1442
116.10	84.62%	83.33%	83.93%	5.0769	0.1846
**116.50**	**84.62%**	**86.67%**	**85.71%**	**6.3462**	**0.1775**
117.50	80.77%	86.67%	83.93%	6.0577	0.2219
122.70	76.92%	86.67%	82.14%	5.7692	0.2663
123.80	73.08%	86.67%	80.36%	5.4808	0.3107
124.00	69.23%	86.67%	78.57%	5.1923	0.355
128.70	65.38%	90.00%	78.57%	6.5385	0.3846
131.10	53.85%	90.00%	73.21%	5.3846	0.5128
137.10	53.85%	93.33%	75.00%	8.0769	0.4945
139.10	46.15%	93.33%	71.43%	6.9231	0.5769
140.20	46.15%	96.67%	73.21%	13.8461	0.557
142.10	38.46%	96.67%	69.64%	11.5385	0.6366
143.90	34.62%	96.67%	67.86%	10.3846	0.6764
144.19	30.77%	96.67%	66.07%	9.2308	0.7162
150.27	26.92%	96.67%	64.29%	8.0769	0.756
156.10	23.08%	96.67%	62.50%	6.9231	0.7958
160.40	19.23%	96.67%	60.71%	5.7692	0.8355
162.20	11.54%	96.67%	57.14%	3.4615	0.9151
169.27	11.54%	100.00%	58.93%	0.8846	
179.00	7.69%	100.00%	57.14%	0.9231	
214.51	0.00%	100.00%	53.57%	1	

LR+, positive likelihood ratio; LR−, negative likelihood ratio. Bold values, optimal cutoff point with corresponding performance metrics.

### 3.9 Clinical impact and utility of the model

Furthermore, the clinical impact and utility of the model were evaluated by DCA. In the DCA curves, the *Y*-axis represents the net benefit, while the *X*-axis indicates the threshold probability.

**Figure 8:**
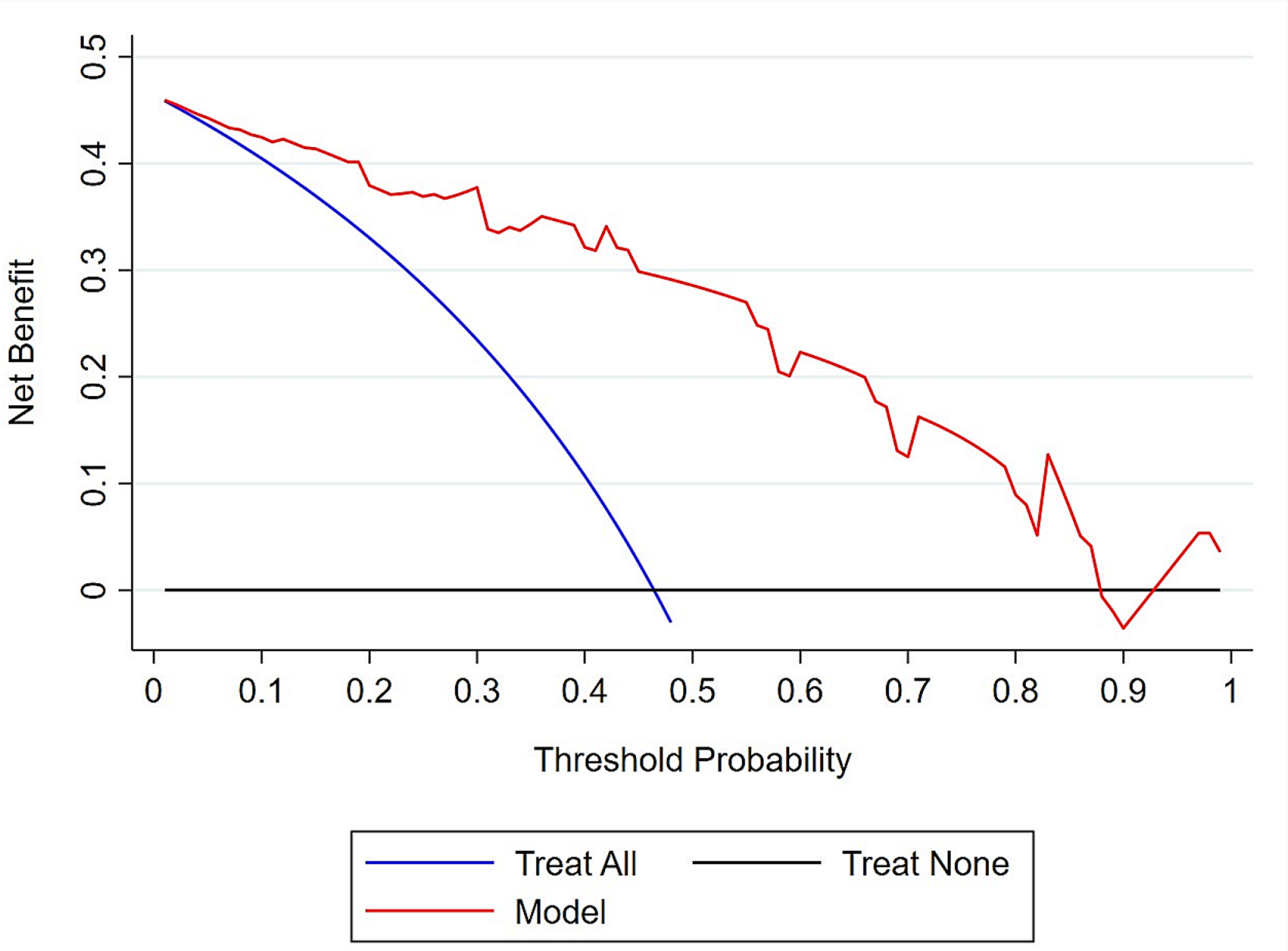
Decision curve analysis plot to demonstrate the clinical usefulness of the model in predicting MCI. The bold red line represents the net benefit of decision making based on the model at different threshold probabilities; the blue line represents the net benefit of treating all subjects with MCI; the black line represents the net benefit of the ‘treat none’ scenario.

The net benefit of the model for each threshold probability was compared with two extreme scenarios: “treat all” and “treat none.” Thus, the DCA indicated that decision-making based on the model yields a higher net benefit for each threshold probability compared to the two extreme situations (Figure 8).

## 4 Discussion

Dementia is a heterogeneous clinical condition that is orchestrated by a wide range of demographic, health-related, and biological factors. Vision impairment has also been recognized as a potential predictor that increases the risk of dementia in older adults, through limiting physical activity and social engagement (Shang et al., 2021; Lee et al., 2021). Specifically, retinal diseases such as AMD, glaucoma, and diabetic retinopathy have shown a significant association with an increased risk of all-cause dementia (Feng et al., 2023). Recently, early retinal structural and vascular changes, prior to the onset of clinically detectable retinal pathologies associated with AD and MCI, have been identified as useful biomarkers that may signal central neurovascular changes underlying early cognitive decline. However, they have not yet been applied in clinical practice (Ge et al., 2021). Following a rigorous model development and validation process, this study introduces a user-friendly nomogram that leverages non-invasive visual and retinal biomarkers for the early identification of cognitive impairment among ophthalmic patients. The nomogram incorporates central MRT, outer nasal MPD, and contrast sensitivity and demonstrates excellent discriminative ability in both the training and testing datasets (AUC: 0.896) to differentiate between individuals with MCI and those with normal cognitive function among ophthalmic patients aged 60 and above, as evidenced by bootstrap validation. Clinical decision-making based on the model, as assessed by DCA, suggests a potential net benefit; however, these findings remain preliminary. Both retinal and visual features exhibited good diagnostic performance, with a discriminative power of 0.78–0.8. These findings align with previous research indicating the potential of contrast sensitivity (Risacher et al., 2013; Elvira-Hurtado et al., 2023), macular retinal thickness features (Almeida et al., 2019; Salobrar-García et al., 2019; Cunha et al., 2016), and retinal vascular features (Chua et al., 2020) as biomarkers for cognitive impairment. Combining central MRT and outer nasal MPD also achieved a discriminative power of 0.84 with an accuracy of 75%, which is similar to the performance of retinal image-based deep learning model combining vascular features of the SVL, deep vascular layer, and choriocapillaris (Hao et al., 2024), OCT images and or retinal photograph (Shi et al., 2024; Gao et al., 2023; Zhang et al., 2021), and ganglion cell-inner plexiform layer thickness with OCTA images (Wisely et al., 2024). Our study enhanced the ability to detect retinal features by complementing them with visual function. Decrease in contrast sensitivity has been associated with AD and MCI (Risacher et al., 2013; Risacher et al., 2020).

The retina, as an extension of the central nervous system, shares structural and vascular traits with the brain, reflecting neural changes in neurodegenerative diseases (London et al., 2013). Importantly, identification of Aβ plaques in the postmortem retina of AD patients has been considered a significant advancement (Koronyo-Hamaoui et al., 2011; Lee et al., 2020; Koronyo et al., 2017), driving a special interest in *in vivo* retinal imaging. Thus, several studies have shown a significant reduction in total macular thickness associated with cognitive decline (Kim and Kang, 2019; Giménez Castejón et al., 2016; Cunha et al., 2017; Cunha et al., 2016; Hao et al., 2023; Ito et al., 2020; Kao et al., 2023). Cognitive scores positively correlate with total macular thickness (Kim and Kang, 2019; Zhao et al., 2023; Mei et al., 2021), with strong correlation in the superior and temporal quadrants. This relationship has been further substantiated by linking the perifoveal retinal thickness to the hippocampal volume (Tao et al., 2019). Specifically, a positive correlation between total retinal thickness and hippocampal volume is more predominant in MCI (Chen et al., 2023). Consistent with previous findings, this study also observed a significant association between macular thickness in central, temporal, and inferior areas and cognitive decline after adjusting for age.

The neurovascular unit of the brain maintains the integrity of the blood–brain barrier and regulates cerebral blood flow, thereby maintaining normal brain function (Yu et al., 2020). Disruption of the neurovascular unit impairs oxygen and nutrient supply, as well as the clearance of neurotoxic substances, such as β-amyloid, resulting in the expression of amyloid precursor protein, capillary hypoperfusion, neurofibrillary tangle formation, neuroinflammation, neuronal damage, and cognitive deficits (Ahmad et al., 2020; Kisler et al., 2017; Nelson et al., 2016; Shabir et al., 2018). Neurodegenerative diseases that cause cognitive decline are associated with reduced neurovascular coupling (Gao et al., 2023) and decreased cerebral blood flow (Li et al., 2023). Similarly, many studies have documented retinal vascular alterations in the posterior pole associated with AD and MCI (Criscuolo et al., 2020; Chua et al., 2020; Fang et al., 2021; Hu et al., 2023; Jiang et al., 2023; Jiang et al., 2018). Retinal vascular changes have been linked to the high burden of retinal amyloid, in which a severe decrease in retinal vascular zonula occludens-1 and claudin-5 correlated with abundant arteriolar Aβ40 deposition in subjects with MCI and AD (Shi et al., 2023). Retinal claudin-5 deficiency has shown a significant association with cerebral amyloid angiopathy, while vascular zonula occludens-1 defects have been linked to cerebral pathology and cognitive decline. This study also detected a significant association between perfusion density in the macular area and cognitive impairment, suggesting an underlying neurodegenerative process. This evidence of altered retinal structural and neurovascular features provides insight into the possibility that the brain and retina may undergo a shared trajectory of neurodegeneration underlying cognitive changes. Altogether, this model comprises predictors that show a trend of association with cognitive changes, although some inconsistencies are present. This evidence suggests that the model is scientifically explainable, as it links the importance of retinal and visual features to cognitive function and their plausible scientific relationship.

Additionally, model interpretability is crucial to the model’s clinical utility. In routine clinical practice, clinicians easily collect the observed value of many predictive variables. However, using the predictors to make a holistic decision about the disease or treatment outcome over time requires a scientific formulation. Nomogram-based models are increasingly considered valuable clinical tools that convert complex statistical models into simple, intuitive graphical risk indicator tools. Given that a nomogram integrates multiple prognostic factors into a single score, nomogram-based prediction provides continuous risk scores, allowing for precise stratification into personalized risk categories, compared with traditional clinical staging of disease. This tool is commonly used in tracking chronic diseases, particularly in the management of cancer (Vernerey et al., 2016; Liu et al., 2022). This study uses readily extractable predictors, without requiring further feature generation, which may ensure technical efficiency and model utilization feasibility. The scoring is intuitively simplified with a dynamic nomogram app, enabling rapid, efficient classification of the target population. Moreover, the net benefit analysis indicated that the model yields a higher net benefit across all threshold probability ranges. Thus, the net benefit suggests that the consequences of decision-making based on the model were cost-effective, despite no economic evaluation having been conducted.

Although the clinical demographic profiles of ophthalmic patients vary with eye care models, the model may target a high-risk segment of patients who are readily accessible for opportunistic screening.

This approach might complement existing cognitive assessments, but decision-making based on the nomogram should be carefully contextualized within broader clinical evaluation. Moreover, because the model was developed with a small sample size, there may be a risk of overfitting and limited generalizability, despite adherence to the clinical prediction model development rule (Stiell and Wells, 1999) and the use of a rigorous variable selection method. Following statistically proven sample size estimation methods such as Riley’s approach might decrease random error (Riley et al., 2019; Riley et al., 2020). Moreover, the retrospective power analysis obtained from this study may also facilitate prior sample size estimation in future clinical studies.

The model was internally validated with good performance, yet external validation with independent, larger datasets with multicenter cohorts is imperative to establish generalizability and clinical applicability. Given these limitations, this nomogram-based risk-scoring tool should currently be regarded as a proof-of-concept that requires further validation before clinical application.

Additionally, MCI is a heterogeneous clinical entity with a complex trajectory, orchestrated by demographic, health-related, and biological factors, so that some progress to dementia, others remain stable or revert to normal (Aerts et al., 2017). Modifiable risk factors such as smoking, obesity, depression, and excessive alcohol consumption have a significant role in dementia prediction and the management process (Blotenberg et al., 2025). This model has not incorporated these modifiable risk factors. Integrating such factors may substantially enhance predictive accuracy for stratifying MCI into progressor and non-progressor phenotypes. Hence, longitudinal studies are warranted to determine potential predictors of MCI progression.

Furthermore, although MMSE has demonstrated a sensitivity of 60% and a specificity of 80–87% (Tariq et al., 2006; Kaufer et al., 2008; Saxton et al., 2009), relying on a single global screening tool may therefore lead to misclassification bias. Thus, comprehensive domain-specific cognitive assessments, including a thorough history and functional assessment, and conventional diagnostic clinical criteria, are important to increase accuracy (Petersen, 2016; McCarten, 2013; Markwick et al., 2012).

## 5 Conclusion

The study developed a simplified diagnostic prediction model that utilizes an intuitive nomogram scoring system, integrating both retinal features and visual function parameters. The model demonstrated relatively good performance and net benefit across all ranges of threshold probabilities, which might complement visual and neurovascular evidence with cognitive tests. Using a well-validated model trained on a large dataset, in conjunction with cognitive tests, might support early detection of MCI in the ophthalmic setting. Thus, further external validation is essential prior to clinical application and generalization.

## Data Availability

The original contributions presented in the study are included in the article/supplementary material, further inquiries can be directed to the corresponding authors.

## Footnotes

1
https://glmnet.stanford.edu

2
http://www.stats.ox.ac.uk/pub/MASS4/

3
https://hbiostat.org/R/rms/

4
https://cran.r-project.org/bin/windows/base/
